# Automatic Apple Detection and Counting with AD-YOLO and MR-SORT

**DOI:** 10.3390/s24217012

**Published:** 2024-10-31

**Authors:** Xueliang Yang, Yapeng Gao, Mengyu Yin, Haifang Li

**Affiliations:** 1 College of Computer Science and Technology (College of Data Science), Taiyuan University of Technology, Jinzhong 030600, China; yangxueliang0448@link.tyut.edu.cn (X.Y.); yinmengyu0743@link.tyut.edu.cn (M.Y.); 2 College of Computer Information Engineering, Shanxi Technology and Business University, Taiyuan 030006, China

**Keywords:** apple counting, YOLOv8, BoT-SORT, multiple-object tracking, appearance features

## Abstract

In the production management of agriculture, accurate fruit counting plays a vital role in the orchard yield estimation and appropriate production decisions. Although recent tracking-by-detection algorithms have emerged as a promising fruit-counting method, they still cannot completely avoid fruit occlusion and light variations in complex orchard environments, and it is difficult to realize automatic and accurate apple counting. In this paper, a video-based multiple-object tracking method, MR-SORT (Multiple Rematching SORT), is proposed based on the improved YOLOv8 and BoT-SORT. First, we propose the AD-YOLO model, which aims to reduce the number of incorrect detections during object tracking. In the YOLOv8s backbone network, an Omni-dimensional Dynamic Convolution (ODConv) module is used to extract local feature information and enhance the model’s ability better; a Global Attention Mechanism (GAM) is introduced to improve the detection ability of a foreground object (apple) in the whole image; a Soft Spatial Pyramid Pooling Layer (SSPPL) is designed to reduce the feature information dispersion and increase the sensory field of the network. Then, the improved BoT-SORT algorithm is proposed by fusing the verification mechanism, SURF feature descriptors, and the Vector of Local Aggregate Descriptors (VLAD) algorithm, which can match apples more accurately in adjacent video frames and reduce the probability of ID switching in the tracking process. The results show that the mAP metrics of the proposed AD-YOLO model are 3.1% higher than those of the YOLOv8 model, reaching 96.4%. The improved tracking algorithm has 297 fewer ID switches, which is 35.6% less than the original algorithm. The multiple-object tracking accuracy of the improved algorithm reached 85.6%, and the average counting error was reduced to 0.07. The coefficient of determination $R^{2}$ between the ground truth and the predicted value reached 0.98. The above metrics show that our method can give more accurate counting results for apples and even other types of fruit.

## 1. Introduction

With the continuous development of smart agriculture, the importance of fruit counting in the management of agricultural production is increasing, and it has become an indispensable management tool for agricultural producers. At present, the fruit-counting method is widely used to predict fruit yields [1]. Of course, in addition to the ability to estimate orchard production, fruit counting can also be used to help farmers with fruit thinning. Most fruit counting in orchards is currently based on the multiple-object tracking problem of Tracking By Detection (TBD). The number of apples in the orchard is determined by detecting and tracking the fruit in the video. However, this method tends to be affected by occlusions and light variations as the method is based on computer vision-based object recognition.

### 1.1. Related Work

The method based on TBD can be divided into two stages: object detection and multiple-object tracking. In the stage of multiple-object tracking, the TBD method uses the results of object detection to associate the boundary boxes of multiple targets one by one to complete the tracking of multiple targets. In recent years, deep learning has been used extensively for object detection. By using deep neural network models, it can automatically learn feature representations in images and conduct end-to-end training in large-scale data, thus achieving significant breakthroughs in object detection tasks. Existing object-detection models can be broadly categorized into two groups based on processing modes: one-stage and two-stage. Due to the separation of detection and classification, two-stage structures are highly efficient in terms of accuracy and recall, such as R-CNN [2], SPPNet [3] and Fast R-CNN [4]. The one-stage architecture runs faster but with less accuracy than the two-stage framework. Examples include YOLO [5] and SSD [6]. In recent years, some researchers have used the YOLO system for fruit detection in agriculture. For example, YOLO-v4 [7] and YOLO-v5 [8] are used to identify apples in an orchard.

The object-detection phase is followed by the phase of multiple-object tracking. The direct fruit-counting method is achieved primarily by tracking apples in the video. Multiple-object tracking methods can be divided into separate detector and appearance feature extractor (SDE) methods and combined detector and appearance feature extractor (JDE) methods. The representative SDE method is SORT [9], a simple online real-time tracking method proposed by Bewley. This method uses the object-detection results of Faster R-CNN [10] and uses the IoU (intersection over union) [11] metric and the Hungarian matching algorithm to associate and match targets frame by frame. Due to the low accuracy of tracking based on a single movement feature alone, Ref. [12] further proposed the DeepSORT algorithm based on the SORT method to extract the appearance features of objects using deep neural networks and then re-identify objects by fusing motion features and appearance features. Later, Ref. [13] proposed BoT-SORT based on ByteTrack [14], which ranks top in the results obtained on the MOTChallenge dataset and is a stable method. However, this method of using CNN to extract the appearance features of the object can lead to slower tracking. Moreover, deep networks for re-identification require additional classification datasets to be trained. Ref. [15] propose a combined detector and appearance feature extractor (JDE) approach that unifies object detection and Re-ID networks. However, unlike SDE methods, JDE methods use video data for training, so the costs for data collection and labeling are high.

With the continuous development of smart agriculture, the technology of using video to count fruit in orchards has developed rapidly. Ref. [16] compared the tracking performance of some MOT algorithms in real orchards and concluded that the DeepSORT method can be applied to apple counting. Ref. [17] proposed a mango counting method based on the YOLOv3 detector, 4-dimensional Kalman filter, and Hungarian matching. However, this method uses only the motion characteristics of the target for matching and is too much affected by changes in the external environment. That is why some tracking methods incorporate appearance features during the matching process to minimize ID switching. However, Convolutional Neural Networks (CNN)-based appearance feature matching methods are not applicable to fruit counting due to the absence of an additional re-identification (Re-ID) dataset and the small variation of the same fruit in neighboring frames. Ref. [18] proposed a counting method for apple videos based on the YOLOv3 detector, fruit appearance features, Mahalanobis distance, and IoU measures, using SIFT descriptors and VLAD (Vector of Local Aggregated Descriptors) methods to calculate the appearance similarity of the two objects. However, the performance of this method is not stable, and the speed does not meet real-time requirements. On this basis, Ref. [19] proposed a new multiple-object tracking method using SURF feature descriptors instead of SIFT descriptors to extract target features, which solves the problem of slow SIFT extraction. However, it still fails to solve the problem of the unstable performance of the VLAD method. Ref. [20] proposed a method that uses the YOLOv4-tiny network for training combined with the CSR-DCF algorithm. The method differs from other methods in that it is based on tracking the trunk of a tree and using the relative displacement of the trunk to obtain the trajectory of the fruit. However, this method is not applicable to complex environments where tree trunks are heavily shaded. Ref. [21] improves on YOLOv7 and DeepSORT to realize automatic wheat counting in UAV videos. Ref. [22] also used the improved YOLOv7 as a detector and DeepSORT as a tracker for automatic wheat counting. The difference is that [22] proposed a ’cross-line partitioning counting’ method to avoid double counting. However, both methods additionally require a Re-ID dataset for training the DeepSORT network.

### 1.2. Purpose of the Study

Although scholars have proposed a variety of excellent MOT methods and have proved that they can be applied to fruit counting, in the complex orchard environment, there are various problems resulting in unsatisfactory results. In terms of counting, the tracker of the multiple-object tracking method associates the bounding box with the track frame by frame according to the IoU distance, depth information, or optical flow [23], which often causes ID switching (the same fruit in the two frames has different ids) due to the covering of the fruit and the camera movement, resulting in inaccurate counting. Current fruit-counting algorithms based on TBD methods suffer from the following problems:

- The detector has insufficient detection ability for occluded apples, which is prone to misdetection and omission.
- The trackers currently used for apple counting do not perform well and cannot handle camera movement.
- The performance of the VLAD appearance feature matching method is unstable and easily interfered by the complex environment.

In order to solve the above problems, this paper proposes a multiple-matching–multiple-object tracking method to solve the fruit-counting problem in complex orchard environments. The main contributions of this paper can be summarized as follows:

- We propose the AD-YOLO model as a detector for the TBD method. Based on YOLOv8s, two modules, including the Omni-dimensional Dynamic Convolution (ODConv) and the Global Attention Mechanism (GAM), are introduced to improve the ability to extract feature information from shaded apples. The Soft Spatial Pyramid Pooling Layer (SSPPL) is also designed and applied to realize the effective use of apple features and increase the sensory field of the network.
- We propose the MR-SORT method as a tracker for the TBD method. Using BoT-SORT as the basic tracking method, it can take into account the camera motion state to some extent. Based on BoT-SORT, we use the VLAD method with SURF feature descriptors for real-time apple appearance feature matching. To enhance the robustness of the VLAD method, we propose a validation mechanism.
- This paper designs and implements an apple counting architecture, which can calculate the number of apples appearing in the input orchard video.

### 1.3. Article Structure

The remainder of the paper is structured as follows: In Section 2, we introduce the experimental materials and methods, including the datasets in this paper. We describe our counting methods, including detectors and trackers. In Section 3, we will present the experimental results of this paper and analyze them. In Section 4, we discuss the restrictions of the approach in this paper. Finally, in Section 5, conclusions are given, and some suggestions for future work are made.

## 2. Materials and Methods

**Figure 1:**
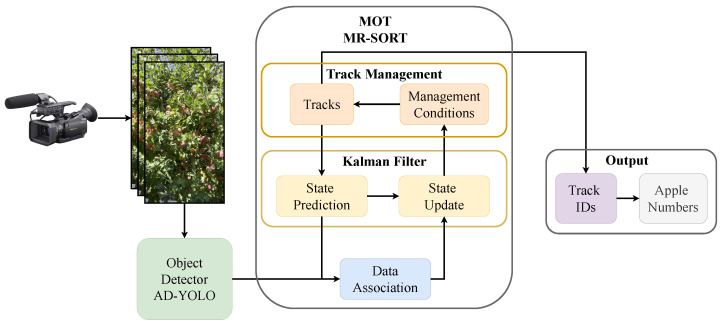
Apple counting channel.

The overall process of apple counting based on object detection and multiple-object tracking is shown in Figure 1. Input a video of the orchard taken by the camera, using an AD-YOLO model to detect and identify apples frame by frame. The detection results are sent to the tracking module, which is responsible for establishing frame-to-frame matching relationships to track each apple. When processing the entire video, each apple in the video is given a different ID value, and the maximum ID value corresponds to the number of apples in the video.

In this section, we will describe the dataset construction process in detail and introduce the model and method proposed in this paper.

### 2.1. Construction of the Datasets

There are two datasets used in this study, both of which are public datasets. The first dataset contains seven videos used for multiple-object tracking and contains the corresponding labeling information. The second contains RGB images used to train the apple detection model, with corresponding annotation information.

#### 2.1.1. Tracking Dataset

The tracking dataset in this paper comes from the public datasets [16], and the second apple tracking database (taken in 2021) is selected. This database consists of 7 videos with a resolution of 1920 × 1080, a frame rate of 30 fps, and a video length of 1 min each. This dataset carries the corresponding annotation information, including frame number, ID, target bounding box, and so on. The public dataset was chosen because the data are open and transparent so that the results can be easily compared. The tracking dataset details are shown in Table 1.

#### 2.1.2. Detection Dataset

**Figure 2:**
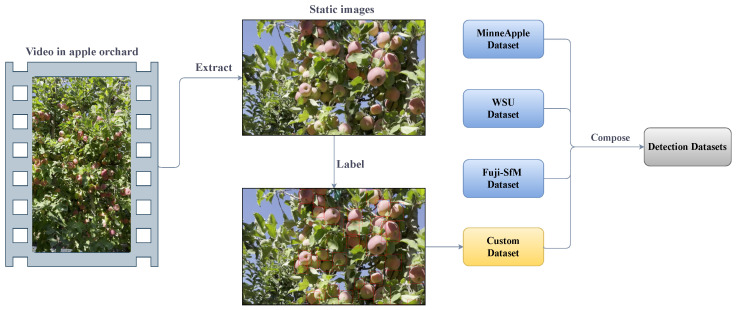
Construction of the detection datasets.

Compared with the single source data, the dataset composed of multi-source data can better deal with the physiological characteristic differences of different kinds of apples and the complex environmental differences of orchards. The detection dataset in this paper is derived from two aspects: on the one hand, three public databases, and on the other hand, part of the tracking dataset mentioned above. The composition of the dataset is shown in Table 2. We extracted 960 images from the above 7 videos at a certain frequency as part of the object-detection dataset. The construction of the detection dataset is shown in Figure 2. The three public databases containing apple RGB images are listed below:

- The MinneApple dataset was collected at the University of Minnesota’s Horticultural Research Center between 2015 and 2016 [24]. The dataset consists of 1000 high-resolution RGB images. Annotation information is given in the form of a mask.
- The WSU dataset [25] was created by the Agricultural Automation and Robotics Laboratory at Washington State University. It contains high-resolution images of apples taken in different lighting conditions. This study uses only the “Crop Load Estimation” data, which contains 238 high-resolution RGB images. The annotation information is given in the form of object-bounding box coordinates.
- The Fuji-SfM dataset [26] was collected in Catalonia, Spain, by the Agricultural ICT and Precision Agriculture Research Group of the University of Leida. It contains 288 high-resolution RGB Apple images. Annotation information is provided in polygon format.

Considering that these common datasets have different annotation formats, we convert the annotation information into the bounding box format. For the MinneApple dataset, the labeling information is given in the form of a Mask. We extract the coordinates of the four vertices (top-left, bottom-left, top-right, and bottom-right) of the bounding box of the Mask data and then compute the centroid coordinates, length, and width of the bounding box. Finally, it is normalized to obtain a uniform labeling format. For the WSU dataset, the labeling information is directly given in the form of a bounding box. Therefore, we directly calculate the center point coordinates and length and width, and then after normalization, we can obtain the unified annotation information. For the Fuji-SfM dataset, the labeling information is given in the format of a polygon. The processing of the labeling information for this dataset is similar to that of the MinneApple dataset, where the coordinates of the four vertices of the bounding box are also obtained first, and then the uniform labeling format can be obtained subsequently.

**Figure 3:**
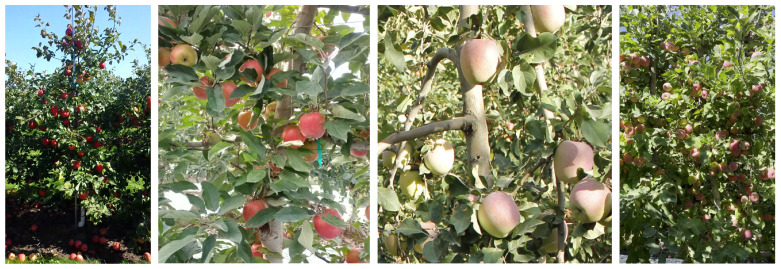
Examples from the four datasets, respectively.

Figure 3 shows some images of the detection dataset, which represent image data from different sources.

### 2.2. Data Augmentation

In the field of image recognition, data augmentation is a very necessary technology. Transforming and expanding the original data increases the diversity of the dataset and makes the training model more adequate. This can effectively reduce the sensitivity of the model to noise and disturbances and improve the generalization capability and accuracy of the model. In this work, we consider data augmentation for horizontal rotation, vertical rotation, brightness changes, contrast changes, saturation changes, Gaussian noise, salt and pepper noise, size changes, and motion blur effects. After augmentation, a total of 22,374 images were divided into training set, verification set, and test set according to the ratio of 8:1:1, and the splitting results were shown in Table 3.

### 2.3. Apple Detection

After a detailed description of the creation of the datasets for this work, the following section briefly describes the structure of the model used to detect apples. Deep learning-based strategies perform better in detection tasks than classical computer vision algorithms, while one-stage methods perform better than two-stage methods in this domain. YOLO is a widely used object-detection algorithm architecture, YOLOv8 [27] is the latest generation version of YOLO, is currently one of the most advanced object-detection algorithms, and is widely used in many computer vision fields. We propose an AD-YOLO apple detection model based on YOLOv8s for detecting apples in video frames.

#### 2.3.1. YOLOv8

The lightweight YOLOv8s was chosen as the base model for this paper. YOLOv8s is a YOLOv8 structure with lightweight parameters. It comprises a backbone network, a neck network, and a predictive output header.

The backbone uses convolution operations to extract features of different scales from RGB images. The backbone network of YOLOv8 retains the Cross Stage Partial Network (CSPNet) idea of the backbone network of YOLOv5, which incorporates gradient changes into the feature map. The backbone network of YOLOv8 consists of five CBS layers, four C2f layers, and one SPPF layer. Each CBS layer consists of convolution, normalization, and activation functions, and the number of stacked C2f modules in each C2f layer is 3, 6, 6, and 3. YOLOv8 retains the spatial pyramid pooling module (SPPF) in YOLOv5 to capture features at different scales, which helps the model to better understand the details of the objects at different sizes and positions and improves the accuracy of object detection.

At the same time, the function of the neck network is to fuse the features extracted from the backbone network. In the neck network, a multi-scale feature pyramid is constructed by combining the advantages of two networks, the Path Aggregation Network (PAN) and Feature Pyramid Network (FPN), to help the network perceive the features of targets at different scales.

The output header layer is responsible for predicting the target category and selecting and detecting the image content with three sets of different-size detection headers. In the prediction header, YOLOv8 uses the Anchor-Free idea to predict the position and size of the target directly without relying on predefined anchor frames, eliminating the process of generating and filtering anchor frames.

#### 2.3.2. ODConv

In the complex orchard environment, the YOLOv8s network makes it difficult to extract all the features of apples due to the occlusion problem, which generates the problem of omission and misdetection of occluded apples. Therefore, this paper introduces the ODConv [28] structure into the backbone network of YOLOv8s, which enhances the ability of the network to extract features of apples from multiple dimensions so that the network can accurately locate the occluded apple areas in complex environments, and improve the detection accuracy of the model. ODConv introduces a multidimensional attention mechanism through a parallel strategy to learn complementary attention in the four dimensions of the convolution kernel space. It has three more dimensions than traditional convolution networks, namely input channel, output channel, and spatial dimension. As an embedded design, ODConv can be plug-and-play and can replace the common convolution structure in the CNN network. The ODConv module formula, defined as Formula (1).

(1) $$y=\left(\alpha_{w1}⊙\alpha_{f1}⊙\alpha_{c1}⊙\alpha_{s1}⊙W_{1}+\ldots +\alpha_{wn}\right.\left.⊙\alpha_{fn}⊙\alpha_{cn}⊙\alpha_{sn}⊙W_{n}\right)*x$$

In Formula (1), $x\in R^{h\times w\times c_{in}}$ and $y\in R^{h\times w\times c_{out}}$ denote the input and output features, respectively; $W_{i}$ denotes the *i*-th convolutional kernel consisting of $c_{out}$ filters; $W_{i}^{m}\in R^{k\times k\times c_{in}},m=1,\cdots c_{out}$ denotes the *m*-th filter of the *i*-th convolutional kernel; ⊙ denotes the multiplication operation in different dimensions along the kernel space; $\alpha_{si}\in R^{k\times k}$ denotes a different attention scalar assigned to the convolution parameters (each filter of the convolution kernel) at the K×K spatial location; $\alpha_{ci}\in R^{c_{in}}$ denotes a different attention scalar assigned to the $c_{in}$ channels of each convolution filter; $\alpha_{fi}\in R^{c_{out}}$ denotes the assignment of different attention scalars to the $c_{out}$ convolutional filters; $\alpha_{wi}$ denotes the scalar used to weight $W_{i}$ by assigning the attention scalar to the entire convolutional kernel, which is the same as the attention scalar in dynamic convolution.

**Figure 4:**
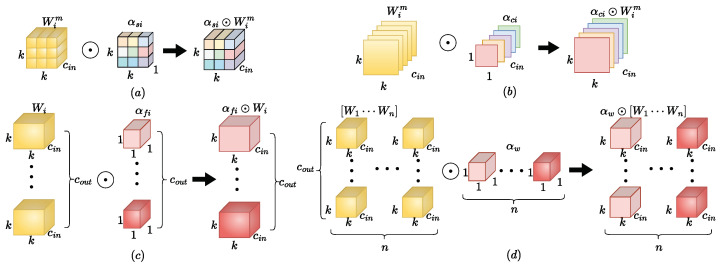
Four types of attention mechanisms in the ODConv network multiplied by the convolution kernel. (**a**) Multiplication of different positions along the spatial dimension, (**b**) multiplication operations along the input channel dimension, (**c**) filtering multiplication operations along the output channel dimension, and (**d**) kernel-wise multiplication along the kernel dimension of the convolution kernel space.

Figure 4 shows four multiplications performed in different dimensions. Multiplying the position, channel, filter and convolution kernel weights by the convolution kernel in that order, the four types of attention are taken into account in this way.

#### 2.3.3. GAM

In complex orchard environments, apples are dense and highly integrated with the background, especially occluding apples. Therefore, in order to enable the network to dynamically select important feature information, reduce feature information dispersion, and improve the detection ability for apples in heavily occluded areas, we introduce the attention mechanism into the backbone network of YOLOv8s. GAM [29] attention mechanism is a kind of attention mechanism that uses both channel attention mechanism and spatial attention mechanism. The GAM process is represented by Formulas (2)–(4).

(2) $$F_{2}=M_{c}\left(F_{1}\right)⊗F_{1}=\mathrm{sigmoid}{\left[K_{1}\cdot \mathrm{ReLU}\left(w_{2}y+b_{2}\right)\right]}^{T}$$

(3) $$y=w_{1}K_{1}^{T}+b_{1}$$

(4) $$F_{3}=M_{s}\left(F_{2}\right)⊗F_{2}=\mathrm{sigmoid}\left[\mathrm{ConvBN}\left(\mathrm{Conv}\mathrm{ReLU}\left(K_{2}\right)\right)\right]$$

where $F_{1}$ is the input feature map, $F_{2}$ is the output feature map of the channel attention sub-module, $w_{1}$, $w_{2}$ and $b_{1}$, $b_{2}$ are the initial weights and bias terms of the multi-layer perceptual machine (MLP), $M_{c}$ is the channel attention function, $F_{3}$ is the output feature map of the GAM attention, and $M_{s}$ is the spatial attention function.

**Figure 5:**
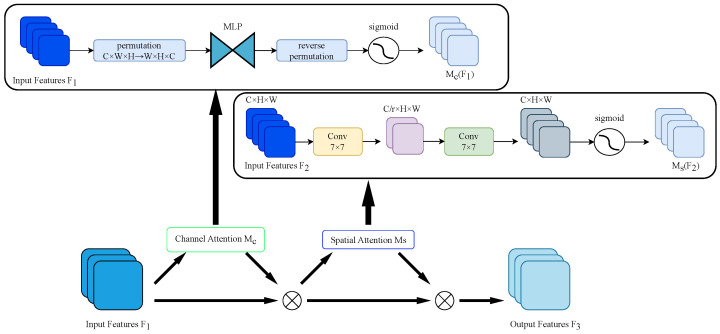
The GAM attention mechanism for the processing of input features in terms of channel and spatial attention.

The processing of GAM for both channel and spatial attention is shown in Figure 5. In terms of channel attention processing, GAM first performs dimension transformation for the input feature map, inputs the dimension transformed feature map to the MLP (Multi-layer Perceptron), then converts it to the original dimension, and finally performs Sigmoid processing and output. In the spatial attention sub-module, two convolutional layers are used for spatial information fusion to focus on spatial information. In summary, the GAM mechanism enhances cross-dimensional interactions by focusing on channel and spatial aspects of information, which can help the model focus on the occluded apples and reduce irrelevant information.

#### 2.3.4. SSPPL

**Figure 6:**
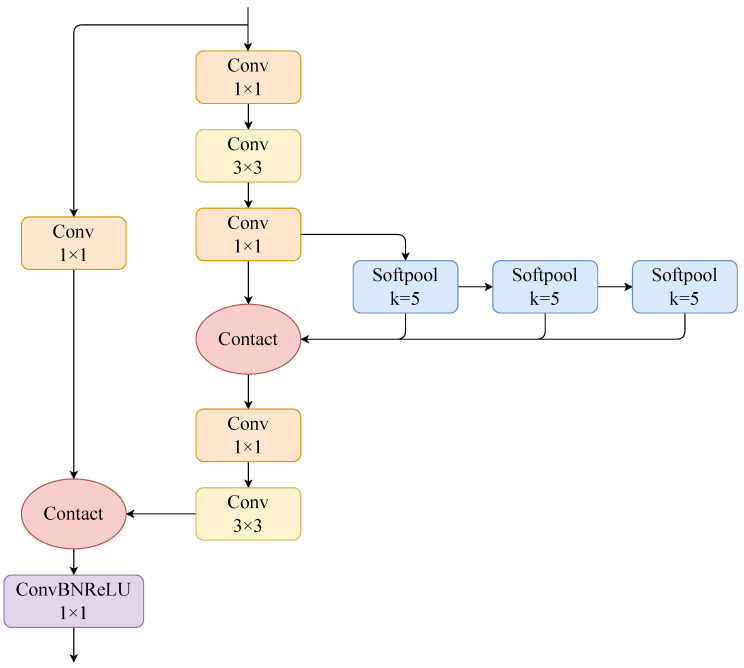
The structure of the SSPPL module.

The spatial pyramid pooling layer in YOLOv8s obtains feature information of different sensory fields by maximum pooling. The feature detail information of apples in the complex orchard environment is not obvious and easily confused with the background information, and the maximum pooling in the SPPF module will lose part of the target detail features, resulting in missing target information. In order to reduce the loss of information in the SPPF module and enhance the ability of the model to fuse the features of different receptive fields, this study draws on the idea of the SPPCSPC [30] module and constructs the SSPPL using Softpool [31], whose structure is shown in Figure 6.

Softpool weights the activation values according to their importance, avoiding the risk of discarding important information by maximal pooling and alleviating the problem of weakening local feature representation by average pooling. Relative to the SPPF module, the SSPPL module adds a sensory field for small targets, using soft pooling instead of maximum pooling, to enrich the contextual information exchange and improve the target detection accuracy of the model while keeping the inference speed almost unchanged.

#### 2.3.5. AD-YOLO

**Figure 7:**
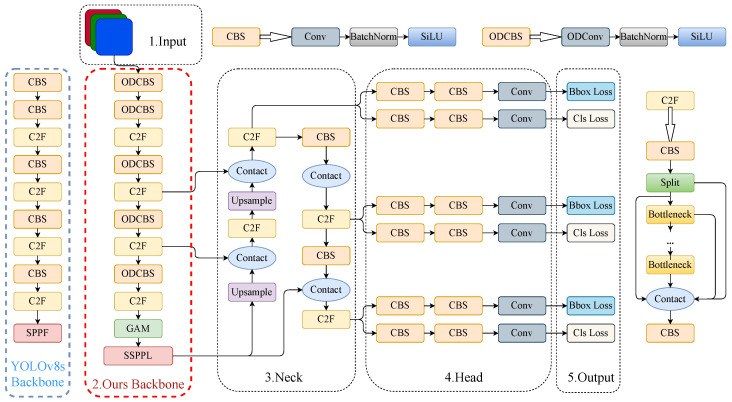
The overall architecture of the AD-YOLO network and our improvements to the YOLOv8s backbone.

This paper presents an improved object-detection model AD-YOLO for fast and accurate detection of apples in natural orchards, whose structure is shown in Figure 7. ODConv is used to replace the common convolution structure in the CBS module of the original network backbone, which enhances the feature extraction capability of the model. In addition, we add the GAM module after the last C2F module of the backbone network to improve network performance by reducing information dispersion and amplifying global interactive representations. At the same time, we replace the SPPF module in the backbone network with the SSPPL module, which enhances the feature fusion capability of the model. The red dotted box in Figure 7 shows all the improvements.

### 2.4. Multiple-Object Tracking

After introducing the object-detection model in this paper, we briefly describe the multiple-object tracking method in this work. BoT-SORT [13] is a multiple-object tracking method with superior performance in the field of pedestrian tracking at present. It can track targets stably when the camera is moving. Based on the BoT-SORT method, this paper proposes an MR-SORT multi-matching algorithm for tracking apples between video frames.

#### 2.4.1. BoT-SORT

BoT-SORT is a superior-performance multiple-object tracking method in the field of pedestrian tracking, which can track the target stably when the camera is moving, and has been used in the field of agriculture. In complex orchard environments, tracking is often inaccurate due to camera instability, so in this paper, the BoT-SORT algorithm with a motion compensation mechanism is chosen as the backbone model of the tracking module. BoT-SORT is divided into two versions, BoT-SORT-ReID and the basic version, which is used in this paper and is referred to as the BoT-SORT algorithm in the following. The basic idea is to use more accurate Kalman filter vectors to predict the high-frame targets and low-frame targets and to combine the camera motion compensation with matching the targets and trajectories.

#### 2.4.2. Appearance Feature Matching

In orchard videos, the frame-by-frame variation of appearance features of the same fruit is negligible. So, we use the SURF descriptor to extract the appearance features of the target and realize feature extraction under the premise of ensuring certain real-time performance. Then, we use the VLAD [32] method to measure the similarity between different targets to realize target rematching based on appearance features.

**Figure 8:**
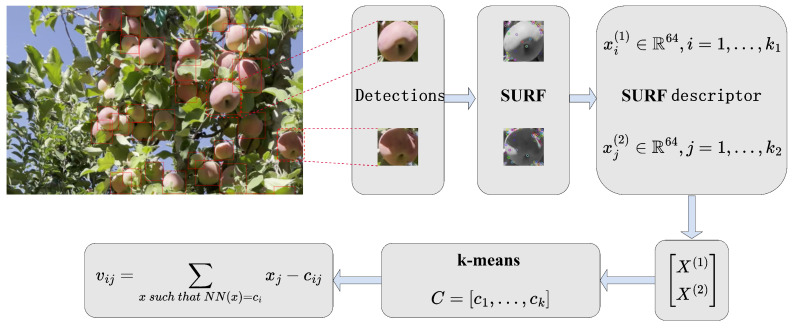
VLAD pipeline: VLAD is used to measure the similarity of the appearance of two objects.

VLAD is an image-matching algorithm, and its process is shown in Figure 8. First, for two images that need to be compared (two apples in the figure), SURF descriptors are used to extract image features. Then clustering algorithm (such as K-means clustering) is used to divide the feature descriptors into several clusters. Next, for each extracted local feature descriptor, the nearest cluster is found and encoded as a vector related to the cluster. Then, all the encoded local feature descriptors are aggregated to generate a global feature representation. Finally, the similarity value between the feature representations of the two images is calculated, and the distance between the two feature vectors is determined using the cosine similarity. The smaller the distance, the more similar the two images are.

#### 2.4.3. Validation Mechanism

**Figure 9:**
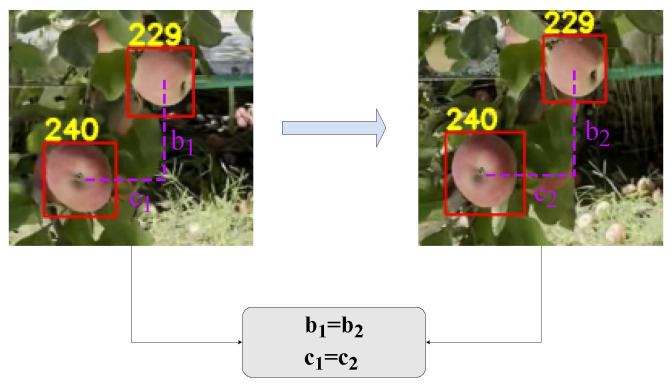
The relative displacement of the same two apples in neighboring frames is the same.

In addition, we found that the performance of the VLAD method is not stable and has some errors. Therefore, we propose a validation mechanism and incorporate it into the VLAD method to reduce the error rate of the VLAD method. The principle of the verification mechanism is that different regions in the same image have the same relative displacement in neighboring frames, as shown in Figure 9. Specifically, this apple is considered to be successfully tracked only if both the apple region and another apple region are successfully matched based on appearance features.

#### 2.4.4. MR-SORT

**Figure 10:**
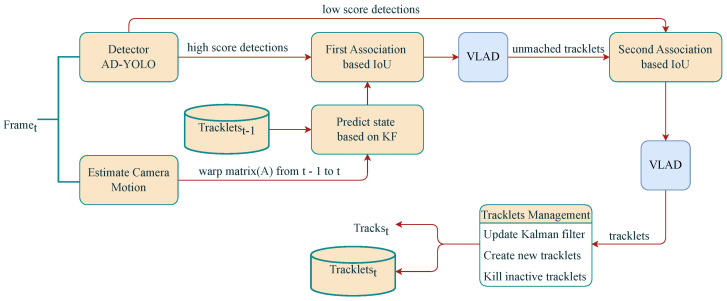
MR-SORT’s tracking process.

In this paper, an MR-SORT method is proposed based on BoT-SORT. Based on BoT-SORT, we add the VLAD module after the first and second IoU-based matching. And using the SURF method in the VLAD module to extract the appearance features of the target. Meanwhile, adding the validation mechanism to the VLAD module to improve the instability of the VLAD method. The process of this method is shown in the Figure 10.

As shown in Figure 10, the main steps of the MR-SORT algorithm are divided into four steps. First, according to the motion state of the camera and the detection result of the previous frame, the Kalman filter is used to predict the new position of each trajectory of the current frame. Second, the Hungarian algorithm is used to match the high-score (high-confidence) box in the current frame with the predicted position according to the IoU and the appearance feature, where the appearance feature matching is based on the SURF descriptor using the VLAD method. Next, IoU and appearance features are used as the similarity criterion to associate the low score box with the unmatched trajectory. Finally, the state of the matched trajectory is updated, a new trajectory is generated for the newly emerged target, the trajectory that has not appeared for a period of time is deleted, and the Kalman filter is updated.

### 2.5. Apple Counting

In this work, the output of the object-detection module is used as the input of the multiple-object tracking module, and the two are combined into one channel for counting apples in orchards, as shown in Figure 1. Using the detection results of the detection module, the tracking module is used to associate the detection boundary box with the trajectory. In multiple matching, fruit appearance features, Mahalanobis distance, and IoU measure are integrated to match the target frame by frame. Finally, the apple counting channel can obtain the number of apples in the video according to the input orchard video.

## 3. Results

This section verifies the accuracy of the proposed algorithm in the video-based apple counting method. The algorithm is divided into two stages: to detect apples in a complex orchard environment by AD-YOLO model; The MR-SORT algorithm is used to track the position of the same apple in different video frames, and the same apple in successive frames is correctly matched.

### 3.1. Experimental Equipment and Parameter Setting

The experiment is carried out in the CentOS Linux operating system with an Intel i9-10900X CPU (Intel, Shanghai, China). The GPU is NVIDIA GeForce RTX 3090 (NVIDIA, Shanghai, China) with 24 GB memory. Pytorch 1.10.0 and CUDA 11.3 as deep learning frameworks. Table 4 shows the detailed configuration information.

The parameter settings of the AD-YOLO model in the object-detection stage and the parameter settings of the MR-SORT method used in the target tracking stage are shown in Table 5.

### 3.2. Evaluation Metrics

#### 3.2.1. Detection Algorithm Evaluation Metrics

This paper evaluates the performance of the object-detection model according to its accuracy and size. The accuracy evaluation indexes adopted in this paper are Precision (P), Recall (R), Average Precision (AP), and an average value of Average Precision (mAP), as shown in Formulas (5)–(8):

(5) $$P=\frac{TP}{TP+FP}\times 100\%$$

(6) $$R=\frac{TP}{TP+FN}\times 100\%$$

(7) $$\mathrm{AP}=\int_{0}^{1}P\left(R\right)dR$$

(8) $$\mathrm{mAP}=\frac{\sum_{i=0}^{N_{C}-1}AP_{i}}{N_{C}}$$

In the above formula, TP (true positive) indicates the correct detection of a positive result; FP (false positive) represents a false positive result, i.e., false detection of a negative result; FN (false negative) represents a false negative result, i.e., false detection of a positive result, and AP represents the average accuracy of each category. The mAP is the average of AP values for all categories. This value can reflect the average detection performance of the model, and the closer it is to 1, the better the detection ability. $N_{C}$ Indicates the number of target types. In this task, the value is 2.

#### 3.2.2. Evaluation Metrics for Counting Performance

The accuracy of the count result can be clearly obtained by using the average accuracy error (comparing the count result with the true value). However, the missing detection behavior of the detector and ID switching can have the opposite effect on the counting accuracy. Therefore, we also used metrics related to the tracking domain to evaluate the performance of the tracking and counting methods.

The evaluation indexes of counting performance adopted in this paper are IDS (ID switching), MOTA (multiple-object tracking accuracy), and MAE (average accuracy error), as shown in Equations (9) and (10):

(9) $$\mathrm{MOTA}=1-\frac{\sum_{t}\left(m_{t}+fp_{t}+mme_{t}\right)}{\sum_{t}\;g\;t}$$

(10) $$\mathrm{MAE}=\frac{1}{m}\sum_{i=1}^{m}\left|\frac{y^{\left(i\right)}-gt^{\left(i\right)}}{gt^{\left(i\right)}}\right|$$

In the above formula, for frame *t*, $m_{t}$ is the number of missed detections; $fp_{t}$ is the number of false detections; $mme_{t}$ indicates the number of ID switches, and gt is the true value. MOTA can be viewed as error rates from three types that can be used to reflect the accuracy of multiple-object tracking.

IDS is the number of ID switches and is used to measure the tracking method’s ability against false matches. MAE is the average accuracy error of all the tested videos, *m* is the number of videos, *y* is the predicted result of the video, and gt is the true number of apples in the video. MAE can be used to reflect the accuracy and robustness of the counting method.

MOTA and IDS represent the performance of multiple-object tracking methods. In addition, we compare the predicted number with the manually calculated true value to evaluate the overall performance of the counting channels.

### 3.3. Apple Detection Results and Analysis

The input image size of the training set, test set, and validation set are all 640 × 640, and the constructed model is trained using the training set.

In order to verify the impact of data augmentation on model performance, we designed comparison experiments. We train the YOLOv8s network using the original dataset and the augmented dataset, respectively, and then compare the results on the test dataset. The results are shown in Table 6. The network trained on the original dataset shows an underfitting phenomenon, and the model accuracy is not up to the requirement. In contrast, the training process of the model trained with the augmented dataset converged with a mAP of 93.3%. It proves that data enhancement can increase the diversity of datasets and improve the performance of the model.

In order to verify the effectiveness and superiority of the GAM attention mechanism added to the backbone network in this paper, we added four different attention mechanisms, GAM, SimAM, EMA, and CBAM, at the same location and used the same experimental environment and parameter configuration to conduct comparison experiments, and the results are shown in Table 7. From the results, it can be seen that the introduction of the GAM mechanism is the most effective, and the recognition time still meets the real-time requirements, although the weight size is improved by 6.7 M.

In order to test the effectiveness of the various improvements proposed in this paper, we conducted ablation experiments in the same environment using the YOLOv8s model (Model 1) as the baseline model, and the results are shown in Table 8.

In Table 8, use ✓ on GAM and ODConv to indicate that this module was added to the backbone, ✓ on SSPPL to indicate that the original SPPF module was replaced with the SSPPL module, and x to indicate that there were no modifications. In Model 2, after the introduction of the attention mechanism GAM, the mAP value of the model is increased by 0.8%, the precision, and recall are both increased by 1.0%, while the number of parameters is increased by 6.7 M. In Model 3, after the addition of the ODConv method in the feature extraction process, the precision rate is increased by 0.5%, the recall rate is increased by 0.4%, the mAP value is increased by 0.4%, and the number of parameters is nearly not increased. In Model 4, after replacing the SPPF module with the advanced SSPPL, the precision rate is increased by 0.7%, the recall rate is increased by 1%, the mAP is increased by 0.7%, and the number of parameters is increased by 6.4 M. In Model 8, when GAM, ODConv, and SSPPL are added to the backbone network at the same time, the mAP increases to 96.4%, and the precision rate increases to 97.4%.

In summary, the detection performance is significantly improved when GAM or SSPPL is added to YOLOv8s, while the mAP value increases slightly after convergence when only ODConv is added. In this paper, Model 8 is named AD-YOLO, which achieves a detection accuracy of 96.4%, laying the foundation for realizing apple tracking and counting.

Then, other SOTA models were trained using the same dataset and the same training strategy, and the parameter results of these models were compared, as shown in Table 9. As can be seen, the AD-YOLO model proposed in this paper enhances the ability to correctly detect apples in complex orchard environments.

### 3.4. Apple Counting Results and Analysis

**Figure 11:**
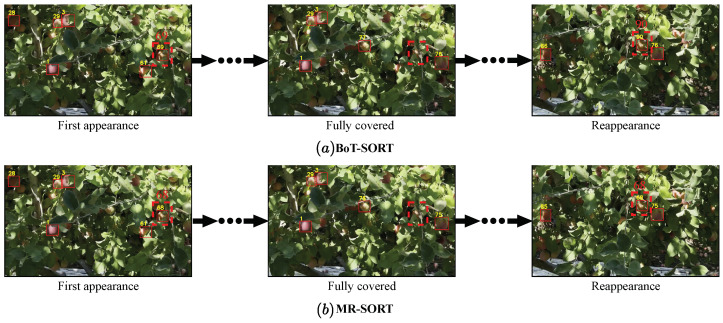
Visualization of one of the countings: comparing the tracking effects of BoT-SORT and MR-SORT using YOLOv8s as a detector.

In order to verify the effectiveness of the MR-SORT method proposed in this paper for tracking counts on orchard videos, we use YOLOv8s as the detector, and the tracking results of the BoT-SORT algorithm and the MR-SORT algorithm are shown in Figure 11. When using the BoT-SORT algorithm, the apple numbered 69 in frame 47 of the video disappears in frame 51 due to occlusion. However, when it reappeared at frame 68, it was incorrectly recognized as a new apple and was given the number 90, whereas when using the MR-SORT algorithm of this paper, when this apple reappeared after being completely occluded, it was successfully recognized as apple number 68 without ID switching.

**Figure 12:**
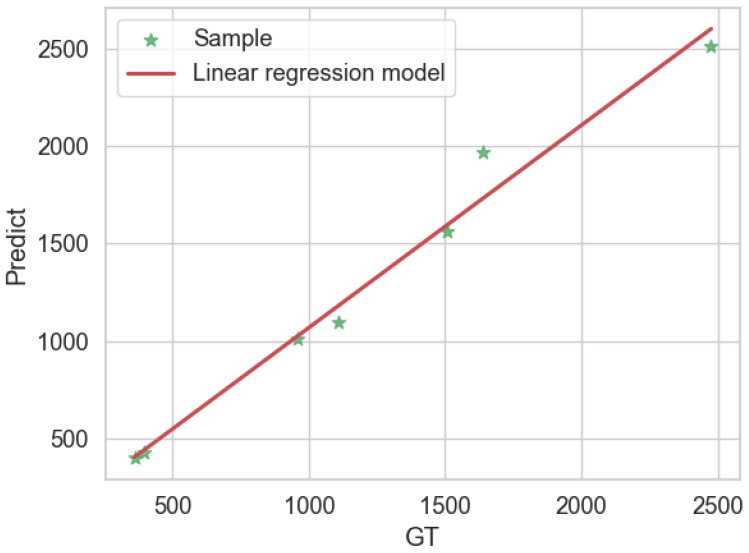
Apple count results for 7 test videos.

Seven video counting results based on the AD-YOLO and MR-SORT methods are shown in Figure 12, and it can be seen that the predicted value and the manually measured value basically fit. Each point represents a video sample, and the straight line represents the linear regression model with the equation y=1.04x+28.24 and the coefficient of determination $R^{2}$ of 0.98. All these indicators show that our method has good performance.

In this paper, counting results and evaluation indexes of tracking algorithms are used to assess the performance of counting methods. We conducted ablation experiments to verify the performance of our detector and tracker, and the results are shown in Table 10. The MR-SORT algorithm tracked better than the BoT-SORT algorithm when using the same detectors (YOLOv8s or AD-YOLO). Similarly, the AD-YOLO model detects targets that are tracked better than the YOLOv8s model when using the same tracker (BoT-SORT or MR-SORT). Therefore, improvements to both the detector and the tracker enhanced the tracking effectiveness and reduced the counting error.

There have been some studies on fruit-counting algorithms based on deep learning and multiple-object tracking. In order to verify the performance of the apple counting algorithm proposed in this paper, the existing related studies are compared with the algorithm in this paper, and the results are shown in Table 11. Compared with other counting algorithms, this paper’s algorithm has advantages in multiple-object tracking accuracy, number of ID switches, and average counting error, and the final predicted value obtained is closer to the true value.

## 4. Discussion

From the above results, it can be seen that our improvement for YOLOv8s has achieved some results and improved the detection accuracy. By adding the GAM structure to the backbone network, we make the model focus more on important feature information, which in turn improves the mAP by 0.8%. Then, the ODConv structure is applied to the network to enhance the feature extraction ability of the model for occluded apples, which in turn improves the mAP by 0.4%. Finally, we designed and applied SSPPL to increase the sensory field of the network, and the mAP was improved by 0.9%. Compared to other SOTA models, our model improves the detection accuracy while ensuring real-time performance. Therefore, in complex environments, our model has an advantage in accuracy.

When using YOLOv8s as a detector, our proposed method outperforms the method using BoT-SORT, with a 0.5% improvement in MOTA metrics, a 28% decrease in IDS, and a 0.03 decrease in MAE. This is because BoT-SORT fails to take into account the appearance features of the target and matches using only the target’s motion features. We added appearance feature matching to the matching process to improve the accuracy of tracking. When using BoT-SORT as a tracker, our proposed method outperforms the method using YOLOv8s, with a 0.7% improvement in MOTA metrics, an 11% decrease in IDS, and a 0.08 decrease in MAE. This is because target detection is the basis of apple counting, and our AD-YOLO model has a higher detection accuracy. Meanwhile, our constructed apple counting algorithm outperforms other counting algorithms in terms of MOTA, IDS, and MAE. This shows the superiority of the method proposed in this paper in tracking and counting.

Although the overall performance has been improved, there are still limitations of the proposed method, which can be discussed in three application scenarios: more complex orchard environments, micro-machine applications, and yield prediction.

Currently, the performance of the apple counting model is still mainly limited by the complex orchard environment. Although the proposed counting method achieved superior results in this study, the performance is likely to degrade to some extent if applied to more complex orchard environments. This is because the complex orchard environment largely affects the accuracy of target detection and the robustness of the tracking method. With the continuous development of deep learning technology, detectors, and trackers that are more suitable for complex environments will appear in the future.

For the application of miniature machines, the overall size of the model should be considered more. The objective of this study is to optimize YOLOv8s for the inspection of apples in complex orchard environments, with a particular focus on shaded apples. Conversely, this process also results in an expansion of the model’s size, which presents a challenge for the deployment of the model in a lightweight manner. Subsequently, the model can be made lightweight while maintaining accuracy using lightweight techniques such as knowledge distillation.

For the application of apple counting algorithms to yield prediction, some researchers would like to predict yields down to the kilogram rather than just predicting the number of apples. Obviously, a system that predicts apple fruit diameter and weight based on that diameter is also needed, and this is a future direction for research.

## 5. Conclusions

In this paper, an AD-YOLO object-detection model and MR-SORT tracking module are proposed to realize the direct counting method of orchard apples using mobile phone videos.

In terms of apple detection, to enhance the robustness of the apple detection model, apple images from multiple sources from different growing environments were used as datasets. We introduced ODConv and GAM structures in the backbone network of YOLOv8s to improve the feature extraction capability of the model for occluded apples. At the same time, we designed the SSPPL structure to improve the ability of the network to fuse features at different scales. The composition of the YOLOv8s model, GAM, ODConv, and SSPPL mechanism can better extract apple features and improve detection accuracy. The results show that the accuracy rate of apple detection is 97.4%, the recall rate is 92.1%, and the mAP value is 96.4%, which reduces the wrong detection and missing detection in the complex orchard environment.

In terms of object tracking, we use the VLAD algorithm with SURF feature descriptors to achieve appearance feature matching and use a validation mechanism to enhance the robustness of VLAD. So, the BoT-SORT algorithm improves the model’s ability to match the same apple during tracking and achieves better tracking results. The results show that the MOTA metric of the multiple-object tracking algorithm reached 85.6%, and a 35.6% drop in IDS to 538, improving the accuracy of apple counting.

For apple counting, we designed an automatic apple counting pipeline for the automatic counting of apple orchard videos. In the extracted orchard video, the average accuracy error (MAE) of this counting method is only 0.07, the coefficient of determination $R^{2}$ is 0.98, and the predicted value basically fits the ground truth.

The following points are made regarding future research directions and possible improvements. First, with the continuous development of deep learning technology, detectors and trackers with superior performance may appear in the future, which can largely improve the accuracy and speed up the apple counting method. Second, in the future, more accurate yield forecasting results should be obtained, not just in terms of the number of fruit obtained, but down to the kilogram. It is possible to use an RGB-D camera to obtain information about the distance of the fruit, which in turn leads to the diameter of the fruit. The weight of the fruit is then fitted to the fruit diameter. The sum of the weight of each fruit is the yield of the orchard. Finally, the need for lightweight of the entire counting model should also be considered for easy deployment in microdevices. Lightweighting of the model is also a key future research focus, including knowledge distillation, channel pruning, and so on.

## Figures and Tables

**Table 1:** Tracking dataset information.

Video	Resolution	FPS	Number of Apples
1	1920 × 1080	30	1509
2	1920 × 1080	30	2475
3	1920 × 1080	30	959
4	1920 × 1080	30	1637
5	1920 × 1080	30	1109
6	1920 × 1080	30	397
7	1920 × 1080	30	361
Total			8447

**Table 2:** The composition of detection dataset.

Data Source	Data Content
MinneApple	1000 high-resolution RGB images
WSU	238 high-resolution RGB images
Fuji-SfM	288 high-resolution RGB images
Custom datasets	960 images were captured from 7 tracking data videos at a certain frequency

**Table 3:** The composition of detection dataset.

Data Source	Train	Valid	Test	Total
MinneApple	7200	900	900	9000
WSU	1713	214	215	2142
Fuji-SfM	2073	259	260	2592
Custom	6912	864	864	8640
Total	17,898	2237	2239	22,374

**Table 4:** Detailed configuration information.

Frame	Parameter
Operating System	CentOS Linux release 7.9.2009 (Core)
CPU	Intel(R) Core(TM) i9-10900X CPU @ 3.70 GHz
GPU	NVIDIA GeForce RTX 3090
Model frameworks	Pytorch 1.10.0 and CUDA 11.3
Programming language	Python3.9

**Table 5:** Detailed parameter setting of the model.

Model	Parameter	Value
AD-YOLO	Optimizer	Auto
	Batch_size	32
	Initial Learning Rate	0.01
	Momentum	0.937
	Weight_decay	0.0005
MR-SORT	track_high_thresh	0.5
	track_low_thresh	0.1
	new_track_thresh	0.7
	match_thresh	0.5
	track_buffer	30
	appearance_thresh	0.5

**Table 6:** Impact of Data augmentation on model performance.

Model	Datasets	Precision	Recall	mAP
YOLOv8s	Original dataset	81.9%	73.2%	81.4%
YOLOv8s	Augmented dataset	94.1%	87.4%	93.3%

**Table 7:** Results of comparative experiments on attentional mechanisms.

Model	Attention Mechanisms	Precision	Recall	mAP	Parameters (MB)	Time (ms)
1	None	94.1%	87.4%	93.3%	**11.14**	**1.8**
2	SimAM	94.3%	87.4%	93.5%	11.23	1.9
3	EMA	94.1%	86.9%	93.2%	11.15	1.8
4	CBAM	94.6%	88.0%	93.8%	11.18	1.8
5	GAM	**95.1%**	**88.4%**	**94.1%**	17.81	2.0

**Table 8:** Improved object-detection model Ablation test results.

Model	GAM	ODConv	SSPPL	Precision	Recall	mAP	Parameters (MB)	Time (ms)
1	x	x	x	94.1%	87.4%	93.3%	**11.14**	**1.8**
2	✓	x	x	95.1%	88.4%	94.1%	17.81	2.0
3	x	✓	x	94.6%	87.8%	93.7%	11.16	1.8
4	x	x	✓	94.8%	88.4%	94.0%	17.52	1.8
5	✓	✓	x	95.2%	89.2%	94.5%	17.85	2.0
6	✓	x	✓	97.1%	91.5%	95.9%	24.19	2.2
7	x	✓	✓	96.1%	90.3%	95.2%	17.61	1.9
8	✓	✓	✓	**97.4%**	**92.1%**	**96.4%**	24.28	2.2

**Table 9:** Comparison of detector performance. The performance of our AD-YOLO model is compared with other models in terms of mAP and model size.

Model	Precision	Recall	mAP	Parameters (MB)	Time (ms)
Faster R-CNN	81.9%	73.1%	81.2%	44.6	10.8
MobileNetV3	87.1%	79.7%	87.1%	3.3	1.4
GhostNetV2	88.9%	81.1%	88.5%	2.6	1.4
ShuffleNetV2	83.3%	75.3%	83.1%	**1.2**	**1.2**
YOLOv5s	93.3%	86.5%	92.7%	7.02	1.7
YOLOv7-tiny	91.9%	86.4%	92.0%	6.01	1.5
YOLOv8s	94.1%	87.4%	93.3%	11.14	1.8
YOLOv8m	96.1%	90.0%	95.1%	25.87	2.5
AD-YOLO	**97.4%**	**92.1%**	**96.4%**	24.28	2.2

**Table 10:** Results of apple counting ablation experiment. We verify the effectiveness of our detector and tracker for apple counting.

Framework	Detector	Tracker	GT	Predicted Value	IDS↓	MOTA↑	MAE↓
1	YOLOv8s	BoT-SORT	8447	9771	835	84.4%	0.17
2	AD-YOLO	BoT-SORT		9204	742	85.1%	0.09
3	YOLOv8s	MR-SORT		9488	603	84.9%	0.14
4	AD-YOLO	MR-SORT		8984	**538**	**85.6%**	**0.07**

**Table 11:** Comparison with other counting algorithms.

Framework	GT	Predicted Value	IDS↓	MOTA↑	MAE↓
Algorithm [18]	8447	11,384	10,383	75.2%	0.58
Algorithm [21]		13,055	11,891	71.5%	0.61
Algorithm [19]		10,527	5269	78.7%	0.32
Algorithm [20]		9512	1163	83.4%	0.26
Ours		8984	**538**	**85.6%**	**0.07**

## Data Availability

Data will be made available on request.
